# 158. Diagnostic Yield and Clinical Utility of Broad Range PCR Testing at a Tertiary Children’s Hospital

**DOI:** 10.1093/ofid/ofad500.231

**Published:** 2023-11-27

**Authors:** Jack Schneider, Kirsten Prabhudas-Strycker, Matthew Samaro, Medical Student, Michael Goings, Kagan A Mellencamp, Haseeba Khan, LaKeisha Boyd

**Affiliations:** Indiana University School of Medicine, Indianapolis, Indiana; Indiana University, Indianapolis, Indiana; Indiana University, Indianapolis, Indiana; Indiana University, Indianapolis, Indiana; Indiana University, Indianapolis, Indiana; Indiana University School of Medicine, Indianapolis, Indiana; Indiana University, Indianapolis, Indiana

## Abstract

**Background:**

Broad range PCR testing (BR-PCR) targets highly conserved DNA sequences of bacteria, fungi, or mycobacteria to detect a broad range of organisms in various clinical samples. Given its potential impact in providing timely diagnoses that cannot always be made through conventional testing (CT), we evaluated the diagnostic yield and clinical impact of BR-PCR at our institution.

**Methods:**

We retrospectively evaluated all clinical specimen types obtained for BR-PCR at Riley Hospital for Children from October 2019 to May 2022. Percent positivity (PP) was determined by specific PCR test type (Bacterial/Fungal/NTM/TB), along with median turn-around times (in days) from sample collection. Medical charts were reviewed, and clinical impact of results was determined.

**Results:**

We identified 956 BR-PCR tests sent from 271 specimens collected from 178 patients. Only 14.5% yielded a positive result with a median days-to-result being the longest for fungal PCR at 8.1 days (7.0, 10.1) and TB PCR being the fastest at 7.8 days (6.8, 9.9). Bacterial BR-PCR yielded an overall PP of 42.6% while Fungal BR-PCR was 10.7%. Positivity rates for NTM and TB were 0% and 0.5%, respectively. Bronchial lavage was the most common specimen type (35.5%) with an overall PP of 19.9%. Of the 271 specimens, 245 returned conclusive results from both BR-PCR and clinical testing (CT) for comparison. A clinically significant organism based on CT was identified in 68 specimens (27.8%), 45 of which were confirmed by BR-PCR. 23 (33.8%) were detected by CT but not BR-PCR. 21 clinically significant organisms not detected by CT were identified by BR-PCR, which led to a change in clinical management in 12 instances: new diagnosis (91.7%); or appropriate initiation (91.7%), escalation (25.0%), or de-escalation (25.0%) of antimicrobial therapy.

**Conclusion:**

BR-PCR overall had low diagnostic yield at our institution but was influenced by specimen type. The clinical utility was predominantly seen in immunocompromised patients in which conventional testing was negative. Further data is needed to determine which specimen types and diagnoses will increase the yield and clinical value of BR-PCR and thus, aid in enhancing diagnostic stewardship.

**Disclosures:**

**All Authors**: No reported disclosures

